# Association of *miR-938*G>A Polymorphisms with Primary Ovarian Insufficiency (POI)-Related Gene Expression

**DOI:** 10.3390/ijms18061255

**Published:** 2017-06-12

**Authors:** Sung Hwan Cho, Eun Hee Ahn, Hui Jeong An, Ji Hyang Kim, Jung Jae Ko, Young Ran Kim, Woo Sik Lee, Nam Keun Kim

**Affiliations:** 1 Department of Biomedical Science, College of Life Science, CHA University, Seongnam 13488, Korea; arana006@naver.com (S.H.C.); tody2209@naver.com (H.J.A.); highko@cha.ac.kr (J.J.K.); 2 Department of Obstetrics and Gynecology, CHA Bundang Medical Center, CHA University, Seongnam 13496, Korea; bestob@cha.ac.kr (E.H.A.); bin0902@chamc.co.kr (J.H.K.); happyiran@cha.ac.kr (Y.R.K.); 3 Fertility Center of CHA Gangnam Medical Center, CHA University, Seoul 06135, Korea; wooslee@cha.ac.kr

**Keywords:** single nucleotide polymorphism, microRNA, primary ovarian insufficiency, gonadotropin-releasing hormone receptor, granulosa cells

## Abstract

MicroRNAs (miRNAs) post-transcriptionally regulate gene expression in animals and plants. The aim of this study was to investigate whether polymorphisms in *miR-938* are associated with the risk of primary ovarian insufficiency (POI) and POI-related target gene regulation. We identified the *miR-938*G>A polymorphisms within the seed sequence of mature miRNA and aligned the seed sequence with the 3′ untranslated region (UTR) of the gonadotropin-releasing hormone receptor (*GnRHR*) mRNA, a miR-938 target gene. We found that the binding of *miR-938* to the 3′-UTR of *GnRHR* mRNA was significantly different between normal and variant alleles. Our data suggests that the dysregulation of *miR-938*G>A influences the binding to *GnRHR* and that *miR-938*G>A polymorphisms might contribute to regulation of POI-related target genes.

## 1. Introduction

MicroRNAs (miRNAs) are small, noncoding, single-stranded RNA molecules that form base pairs with target messenger RNA (mRNA) [1]. Previous reports show that miRNAs modulate gene expression by targeting mRNA for deregulation or translational repression [2]. miRNAs are typically composed of about 23 nucleotides and regulate target genes through RNA silencing and post-transcriptional regulation of gene expression [3]. miRNAs have been implicated in the regulation of several biochemical pathways in many eukaryotic organisms [4,5]. miRNAs are transcribed into long precursor transcripts called primary (pri)-miRNAs by RNA polymerase II, and the pri-miRNAs are converted to pre-miRNAs by DROSHA, an enzyme that belongs to an RNase type III family in a complex with DiGeorge Syndrome Critical Region 8 (DGCR8) [6,7]. The pre-miRNA is then exported to the cytoplasm by the exportin5 (Exp5)–ras-related nuclear protein (RAN)-Guanosine-5′-triphosphate (GTP) complex [8]. RAN is a small GTP-binding protein of the RAS superfamily that is essential for the translocation of RNA and proteins through the nuclear pore complex. The Ran GTPase binds Exp5 and forms a nuclear heterotrimer with pre-miRNA [9]. The pre-miRNA undergoes an additional processing step by the RNAse III DICER, and after DICER cleavage an miRNA Duplex is released, a double stranded RNA approximately 23 nucleotides in length. DICER also initiates the formation of the RNA-induced silencing complex (RISC) [10]. RISC is responsible for the gene silencing mediated by miRNA expression and RNA interference (RNAi). miRNAs carry out their biological functions by binding to the 3′-untranslated region (UTR) of target mRNAs, thereby repressing expression. A single miRNA may regulate multiple targets and thus act as a master controller of gene expression.

Recent studies have showed that miRNAs are involved in ovarian pathophysiology including primary ovarian insufficiency (POI) and polycystic ovarian syndrome (PCOS) [11,12]. These findings suggest that miRNAs may be essential for the normal functioning of the reproductive system. POI, also known as premature ovarian failure, is a condition characterized by premature depletion, dysfunction, or lack of ovarian reserves that leads to infertility [13]. POI is biochemically characterized by low levels of gonadal hormones (estrogens and inhibins) and high levels of gonadotropins (LH and FSH) (hypergonadotropic amenorrhea) [14]. Measurement of serum LH is also important. In most cases of spontaneous POI/ Premature ovarian failure (POF), FSH is higher than LH. If autoimmune oophoritis is present, FSH may be only mildly elevated, sometimes below the cutoff of 40 µIU/mL, while LH is markedly elevated [15].

Single-nucleotide polymorphism (SNP) , often abbreviated to SNP, is the most common genetic variant in DNA expression, and the expression of a specific gene may be affected or regulated by its genetic variations [16]. SNPs or mutations in miRNA genes may affect the properties of miRNAs, altering their expression and/or maturation [17]. Sequence variation around the processing sites, and sequence variations in the mature miRNA, especially the seed sequence, may have profound influence on miRNA biogenesis and function [18]. Polymorphisms of pre-miRNAs were first reported in 2005 [19], and several miRNA polymorphism association studies have since been reported [20,21]. Aberrant expression of miRNA has been implicated in numerous disease states, and miRNA-based therapies are being investigated extensively [22,23].

A previous report showed that the miRNA miR-938 is associated with the transforming growth factor (TGF-β) signaling pathway [24]. TGF-β superfamily members exert critical functions in the female reproductive system [25] given the roles of TGF-β and miRNAs in female reproductive physiology, and the miR-938–TGF-β association, we sought to delineate the role, if any, of miR-938 polymorphisms in POI. Polymorphism of *miR-938*G>A (rs12416605) is located seed sequence of mature miRNA. Therefore, we hypothesized that miRNA polymorphisms might, therefore, also be associated with female reproductive diseases, including POI. *miR-938*G>A (rs12416605) is a located seed sequence of mature miRNA. Therefore, we assessed whether the allelic difference of *miR-938* of rs12416605 in regulatory activity is attributable to an altered binding affinity for GnRHR mRNA. In this study, we investigated whether polymorphisms in *miR-938*G>A (rs12416605) were associated with POI in a population of Korean women.

## 2. Results

### 2.1. Genetic Analysis

In this study, we evaluated the relationship between the miR-938G>A polymorphism and POI in a population of Korean women.

Clinical characteristics, including follicle-stimulating hormone (FSH), luteinizing hormone (LH), and estradiol (E2) levels of POI patients and controls, are summarized for comparison in Table 1. The 2 groups were matched in terms of age. The patients with POI showed significantly increased FSH and LH levels and decreased E2 levels compared with the control population (*p* < 0.05).

To examine the relationship between *miR-938* polymorphisms and hormonal levels, we measured the plasma levels of FSH, LH, and E2 from the women harboring the *miR-938*G>A polymorphisms (Table 2). The data show that FSH, LH, and E2 levels were not significantly different between patient and control subjects harboring the GG and GA polymorphisms of *miR-938*G>A (*p* > 0.05). AA genotype did not existed in POI patients and controls in Korean woman. Supplement Table 1 shows the distribution of genotypes in POI patients and control subjects. The miRNA genotype frequencies of POI patients and controls were consistent with expected Hardy–Weinberg equilibrium values. GA heterozygous type was associated with higher risk of POI, compared with the respective wild-type homozygous genotype; however, the odds ratio (OR) of the *miR-938*G>A polymorphism was not significantly different between POI patients and control subjects.

### 2.2. The Impact of miR-938G>A Polymorphisms on Regulation of the 3′-UTR of the Gonadotropin-Releasing Hormone Receptor GnRHR Gene

**Figure 1:**
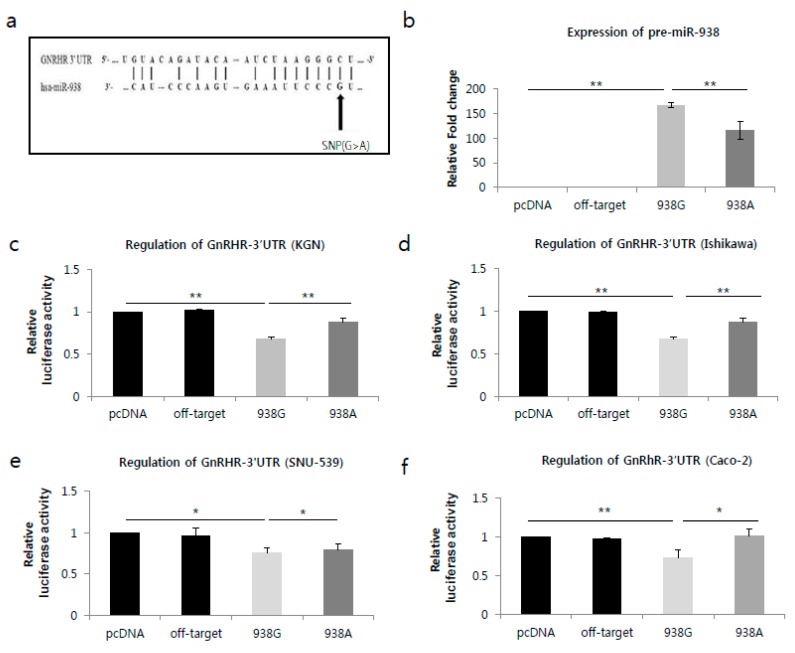
miR-938 targets the GnRHR mRNA through a targeting sequence located at the 3′UTR. (**a**) The miR-938 targeting sequence located in the 3′UTR of GnRHR mRNA. (**b**) miR-938 expression analysis. miR-938 level was detected in cells transfected with the empty pCR3.1 vector, pCR3.1-miR-offtarget, pCR3.1-miR-938-G or pCR3.1-miR-938-A by miRNA quantitative realtime PCR. U6 snRNA was used as the internal control. The relative level of miR-938 was calculated by normalizing miR-938 levels to that of U6 snRNA. ** *p* < 0.05. (**c**–**f**). Dual-luciferase reporter assays were performed to test the interaction of has-miR-938 and its targeting sequence in the GnRHR 3′UTR using constructs containing the predicted targeting sequence (pGL4.13-GnRHR 3′UTR) cloned into the 3′UTR of the luciferase reporter gene. Co-tranfectsion of the luciferase reporter and the miR-938 constructs were carried out in (**c**) KGN, (**d**) Ishikawa, (**e**) SNU-539 and (**f**) Caco-2 cells. The data represent three independent experiments with triplicate measurements of each sample. * *p* > 0.05, ** *p* < 0.05.

We used genetic interaction analysis to predict miRNA target genes using TargetScanHuman (http://www.tragtescan.org). The *miR-938*G>A polymorphism was confirmed to be within the seed sequence of the mature miRNA, which regulates GnRHR expression by binding to the mRNA, as shown in Figure 1a. To experimentally confirm the interaction between *miR-938*G>A, and 3′UTR of GnRHR, we applied reporter gene assays. We cloned fragments of 3′UTR segments of GnRHR (3′UTR is always the same between GNRHR alternative transcripts) into the pGL4.13 expression reporter vector (Promega, Madison, WI, USA). Then, we transfected pGL-4.13-3′UTR (GnRHR) into KGN, Ishikawa, SNU-539 and Caco-2 cell lines with pcDNA3.1-miR-938 or control. All corresponding nucleotides of the target sequences (3′UTR of GnRHR) are the same, While all *miR-938*G>A share the same sequence at positions 1–8 seed sequence except for a SNP position (rs12416605). The binding site included nucleotide at position 2 (miR-938G; black arrow) in Figure 1a.

To investigate the functional impact of the SNP on the expression of *miR-938*G>A, we constructed a *pri-miR-938* expression plasmid under the control of the CMV promoter with either the G or A allele, and transfected the plasmids into a human granulosa cell line (KGN). The expression of *pre-miR-938* with the A allele was significantly lower than that expression with the G allele (*p* < 0.05; Figure 1b). We assessed whether the allelic difference of *miR-938* of rs12416605 in regulatory activity is attributable to an altered binding affinity for GnRHR mRNA. We co-transfected a GnRHR expression construct along with the reporter gene construct containing either the A or G allele of *miR-938* of rs12416605 into human granulosa (KGN), endometrial adenocarcinoma (Ishikawa and SNU-539) and colon adenocarcinoma (Caco-2) cells.

Reporter gene assay was performed under the same treatment conditions. Compared to the off-target control, when GnRHR was co-transfected with the *miR-938*G allele-containing reporter or the *miR-938*A allele-containing reporter, the luciferase activity was significantly decreased (*p* < 0.05; Figure 1c,d) in KGN (*miR-938*G: 34%, *miR-938*A: 14%) and Ishikawa cells (*miR-938*G: 32%, *miR-938*A: 12%). However, the binding capacity was stronger in cell lines transfected with the wild-type G allele compared with those transfected with the A allele in KGN and Ishikawa cells (*p* < 0.05; Figure 1c,d). On the other hand, compared to the off-target control, when GnRHR was co-transfected with the *miR-938*G allele-containing reporter or the *miR-938*A allele-containing reporter, the luciferase activity was not significantly decreased in *miR-938*G and *miR-938*A in SNU-539 cells (Figure 1e). In addition, luciferase activity in *miR-938*A was not higher than with *miR-938*G allele. Then again, compared to the off-target control, when cells were co-transfected with the *miR-938*G allele-containing reporter or the *miR-938*A allele-containing reporter in Caco-2 cells, the luciferase activity was significantly decreased with the *miR-938*G allele only (26%) (*p* < 0.05; Figure 1f).

## 3. Discussion

To date, increasing evidence has supported the roles of microRNAs in reproductive disorders [26,27]. Simultaneously, evidence supporting the role of miRNAs in oocyte maturation and ovarian function has also been accumulating [1,28,29,30,31,32,33]. Based on these recent developments, we sought to investigate whether pre-miRNA SNPs are associated with POI.

GnRHR expression has been detected in reproductive tissues including the ovary, testes, endometrium, myometrium, prostate, breast, and placenta [34]. Unique transcription initiation sites have been characterized in pituitary, ovarian, and placental tissues, which likely explain tissue-specific expression of this transcript. Co-localization of GnRH and its receptor in multiple cell types strongly suggests that GnRH may act in an autocrine/paracrine manner beyond the regulation of gonadotropin secretion [35,36,37]. However, the mechanisms through which GnRHR is regulated by miRNA in humans are yet to be elucidated.

The exact mechanisms still remain unknown. However, several studies have demonstrated that genetic variants in miRNA precursors (pre-miRNA) can affect miRNA expression levels [38,39]. We investigated whether the *miR-938*G>A polymorphisms in *miR-938* affects its binding to the target gene (specifically, GnRHR) mRNA and promotes allele-specific regulation of GnRHR. Our data showed that the expression of *pre*-*miR-938* with the A allele was significantly lower than that with the G allele. Transient expression of GnRHR (3’UTR of GnRHR) greatly reduced the reporter gene activity from *miR-938*G allele in KGN cells. To further explore the possible molecular mechanisms invoked by *miR-938*G>A, we analyzed the binding status of *miR-938*G>A and its target gene. A dual-luciferase reporter assay was used to validate whether *miR-938*G>A directly targeted *GnRHR* mRNA 3′UTR. We observed that the binding between *miR-938*G>A and the 3′UTR of *GnRHR* was significantly different between the wild (G) and variant (A) allele, as indicated by the significantly different luciferase activities. The binding was stronger, as indicated by the lower luciferase activity, in cell lines transfected with wild-type G allele compared to those transfected with the variant A allele in granulosa cells and endometrial cells. In addition, unlike granulosa cells, luciferase activity in miR-938A was not higher than with *miR-938*G allele in the SNU-539 cells. These findings suggest that miR-938 polymorphisms differentially affect GnRHR expression in each cell line. Although we observed a marked differences in luciferase levels in cells co-expressing *miR-938*G>A together with 3′UTR of GnRHR, Western blot analysis is needed to confirm the difference of the translated product (GnRHR protein) in the KGN cells.

An online search of *miR-938* targets using the TargetScan, and miRIAD (http://bmi.ana.med.uni-muenchen.de/miriad/) miriad databases provided a large number of putative mRNA targets. Among them, we focused on GnRHR for further functional analyses of *miR-938*G>A, due to the critical function of GnRHR in reproductive physiology and its key role in. development of clinical strategies to treat reproductive-related disorders [40,41]. The association of *miR-938* with GnRHR expression should also be considered in the context of the association of GnRH agonists with hypothyroidism. Thyroid hormones affect the oocytes at the granulosa and luteal cell level [42]. This is an additional molecular pathway in the miR-938-mediated regulation of GnRHR that warrants further analysis.

Our data suggest that the dysregulation of *miR-938*G>A influences the binding to the 3′UTR of GnRHR, thereby may affecting GnRHR expression. In this context, our data show that although none of the *miR-938* genotypes was associated with POI risk if considered individually, the, GA heterozygous type yielded a higher risk of POI, compared with the respective wild homozygous GG genotype. An increase in GnRHR expression in the immortalized gonadotrope cell line LbT2 was shown to disrupt the FSH response [43]. Therefore, we speculate that the abnormal regulation of GnRHR by G to A substitution in *miR-938* rs12416605 may result in a disruption of the response of the FSH levels. FSH is the primary gonadotropin responsible for the progression of POI [44].

FSH and LH secretion from the gonadotrope is controlled by the hypothalamic decapeptide, GnRH [45]. Acting primarily in the anterior pituitary, GnRH binds to its native high-affinity seven-transmembrane receptor (GnRHR) on the cell surface of the gonadotrope, stimulating signaling cascades that confer the production of these gonadotropins. FSH and LH exert their effects on the ovaries and testes, leading to steroidogenesis and gametogenesis, highlighting their critical role in reproductive function [46].

Due to limited access to ovary samples from patients with POI, however, we could not compare expression levels of miRNAs and target genes according to the miRNA genotypes, which is a major limitation of our study. Although underlying mechanisms of the *miR-938*G>A polymorphisms in the development of POI have not been demonstrated in our study, the data nevertheless suggest that the *miR-938*G>A polymorphisms might contribute to regulation of POI-related target genes and in particular, GnRHR. Further studies of other pre-miRNA polymorphisms in diverse ethnic populations will aid in the understanding of the role of miRNA polymorphisms in POI and POI target-gene regulation.

## 4. Materials and Methods

### 4.1. Ethics Statement

The study protocol was approved by the Institutional Review Board of CHA Bundang Medical Center (ID No. 2010-01-123). All study subjects provided written informed consent to participate in the study. All the methods applied in the study were carried out in accordance with the approved guidelines.

### 4.2. Study Subjects

Blood samples were collected from 143 patients with POI (mean age ± SD, 31.34 ± 4.97 years) and 227 control participants without POI (mean age ± SD, 33.34 ± 5.70 years). All patients were diagnosed with POI (cessation of menstruation before 40 years of age and 2 serum FSH concentration measurements >40 IU/L) at the Department of Obstetrics and Gynecology of CHA Bundang Medical Center from March 1999 to February 2010. Patients with a history of pelvic surgery, cancer, radiation exposure, autoimmune disorder, or genetic syndromes were excluded from this study. The control group consisted of 227 subjects who had regular menstrual cycles and at least 1 live birth. The control group was recruited from CHA Bundang Medical Center. All patients and controls were Korean.

### 4.3. Genotyping

DNA samples from POI patients and controls were extracted using the G-DEX blood extraction kit (iNtRON Biotechnology Inc., Seongnam, Korea). miRNA SNPs were selected using the human genome SNP database (dbSNP; http://www.ncbi.nlm.nih.gov/snp): *miR-938*G>A (rs12416605). Samples were genotyped by PCR-restriction fragment length polymorphism (PCR-RFLP) analysis with the following primers and PCR conditions. *miR-938*G>A polymorphism was detected using primers (forward) 5′-T GGT GCA CTG GGT TCA CCT TTA AGC G-3′ and (reverse) 5′-GTA ATA CCT CTG AGC CTT TGG GGC C-3′ under conditions of initial denaturation at 95 °C for 5 min; 35 cycles of denaturation at 95 °C for 30 s, annealing at 64 °C for 30 s, and extension at 72 °C for 30 s; and final extension at 72 °C for 5 min. PCR products were digested using the restriction enzyme *Hha*I (New England BioLabs, Ipswich, MA, USA).

### 4.4. Construction of miR-938G>A and 3′-UTR of GnRHR Expression Vectors

To create pri-miR-938 G, and pri-miR-938A constructs, genomic fragments (each 522 bp) corresponding to pri-miRNA and its flanking regions were amplified from human genomic DNA (previously determined to have the G or A genotype) and cloned into the vector pcDNA3.1 (under the control of CMV promoter) (Invitrogen, Carlsbad, CA, USA). The sequences of both vectors were confirmed by direct sequencing; the only difference was in the SNP. To create the *miR-938* target gene::luciferase reporter constructs, the relevant fragment of the *GnRHR* gene corresponding to the 3′-UTR region of GnRHR, were amplified and cloned into the pGL4.13-luciferase vector (Promega, Madison, WI, USA). The cDNA was PCR-amplified using the primers forward 5′-GCT CTA GAG CTG GCA CGT CCT TTC TTT CTT-3′ and reverse 5′-TTT GGC CGG CCA AAC AGT CTG GTC CAT CCC TCT C-3′. All constructs were verified by sequencing.

### 4.5. Cell Transfection and Luciferase Assay

Human granulosa (KGN), endometrial adenocarcinoma (Ishikawa and SNU-539) and colon adenocarcinoma (Caco-2) cells were plated at 1 × 10^6^ cells per well in a 6-well plate and transfected 24 h later using Jetprime transfection reagent (Polyplus, France). Each transfection reaction contained 500 ng of 938-G (in pcDNA3.1) or 500 ng of 938-A (in pcDNA3.1), along with 3′-UTR-GnRHR in pGL4.13, and pGL4.75 (normalization control). As controls we used off-target control miRNA (Qiagen, Hilden, Germany). The Dual-Luciferase Reporter Assay System (DLR assay system, Promega, Madison, WI, USA) was used to perform dual-reporter assays on pGL4.13 based reporter systems. DLR Assay System was used to measure luciferase activity of cells co-transfected with 3′UTR-GnRHR vectors and pGL4.73 control vector. Twenty-four hours after transfection, growth medium was removed and cells were washed gently with phosphate buffered saline. Passive lysis buffer (100 μL/well; Promega, Madison, WI, USA) at 100 μL/well was added and the plates were rocked gently for 15 min at room temperature, after which cell lysates were harvested for DLR assay. Cell lysates (10 μL) were transferred in white opaque 96-well plates (Corning Inc., Corning, NY, USA). Firefly and Renilla luciferase activity assays were performed sequentially to the cell lysate in each well. For each luminescence reading, after the injector dispensed assay reagents into each well, a 2 s pre-measurement delay, followed by a 10 s measurement period was employed. Relative luciferase activity was calculated as the ratio of Firefly/Renilla luciferase activity (with 3′-UTR-GnRHR vectors co-transfected with pGL4.73 vector as internal control) to normalize for cell numbers and transfection efficiency.

### 4.6. Statistical Analysis

Differences in the frequency of miRNA polymorphisms between the control and patient groups were assessed using Fisher's exact test and logistic regression model. OR, and 95% confidence intervals (CIs) were calculated. Also, the mean standard deviation (SD) and percentages were determined. Data analysis was carried out using MedCalc version 12.1.4 (MedCalc, Ostend, Belgium) and GraphPad Prism 4.0 (GraphPad, San Diego, CA, USA) software packages. We used genetic interaction analysis to predict miRNA target genes using TargetScanHuman (http://www.targetscan.org).

## Figures and Tables

**Table 1:** Clinical variables of Korean primary ovarian insufficiency (POI) patients and control subjects.

Characteristics	Controls (227)	POI Patients (143)	*p*
Age (years) mean ± SD (range)	33.34 ± 5.70 (23–43)	31.34 ± 4.97 (21–43)	NS
FSH (mIU/mL) mean ± SD	8.12 ± 2.85	66.46 ± 14.11	<0.0001
LH (mIU/mL) mean ± SD	3.32 ± 1.761	26.23 ± 10.68	<0.0001
Estradiol (pg/mL) mean ± SD	26 ± 14.75	7.93 ± 2.59	<0.0001

Abbreviations: SD, standard deviation; NS, not significant; FSH, follicle-stimulating hormone; LH, luteinizing hormone; POI, primary ovarian insufficiency; *p* values were calculated using the t test.

**Table 2:** FSH, LH and E2 levels in control subjects and POI patients with different miRNA polymorphic genotypes.

	FSH (mIU/mL) Mean ± SD		LH (mIU/mL) Mean ± SD		E2 (pg/mL) Mean ± SD	
	Control (227)	Case (143)	Control (227)	Case (143)	Control (227)	Case (143)
*miR-938*G>A						
GG	8.16 ± 8.49	62.06 ± 13.31	3.36 ± 3.28	26.44 ± 10.55	25.43 ± 205.77	7.86 ± 2.50
GA	7.36 ± 1.36	65.46 ± 24.83	2.44 ± 0.75	22.90 ± 12.96	35.83 ± 381.45	8.86 ± 3.87
AA	-	-	-	-	-	-
*p*	0.743	0.511	0.25	0.465	0.093	0.692

Data shown are the mean ± SD. Abbreviations: SD, standard deviation; FSH, follicle-stimulating hormone; LH, luteinizing hormone; E2, estradiol; POI, primary ovarian insufficiency.
